# A Curious Case of Ear Necrosis Salvaged by a Composite Temporalis Flap: Conchal Cartilage Graft Reconstruction

**DOI:** 10.7759/cureus.15653

**Published:** 2021-06-15

**Authors:** Leon Alexander, Honey Chacko

**Affiliations:** 1 Plastic & Reconstructive Surgery, Sheikh Khalifa Medical City, Abu Dhabi, ARE; 2 Dermatology, Ahalia Hospital, Abu Dhabi, ARE

**Keywords:** ear hemangioma, sclerotherapy, ear necrosis, nicolau syndrome, ear reconstruction, vascular tumor

## Abstract

Hemangiomas are commonly encountered benign vascular tumors in clinical practice. They are easily diagnosed clinically, but it is essential to know atypical and rare varieties of these tumors to avoid confusing them with vascular malformations. The traditional approach in managing hemangiomas has been a “wait and watch” policy as most of these lesions undergo spontaneous regression with time. There are multiple treatment modalities in managing these lesions, but with specific indications for each of them. We report a case of Nicolau syndrome following injection sclerotherapy for a residual ear hemangioma, which lead to necrosis and total loss of skin and cartilage. However, the full-thickness defect in the ear was restored with a composite temporalis fascial flap, conchal cartilage graft, and split skin graft reconstruction. A detailed literature review of the presentation and management of this vascular tumor is discussed with a special emphasis on avoiding complications and maximizing patient outcomes.

## Introduction

Hemangiomas are the most common benign vascular tumors of infancy [1]. They are not congenital tumors, appear after birth, and undergo rapid growth during infancy followed by a slow, gradual regression during childhood. Thus, there are three stages in its life cycle: the proliferating phase (0-1 year of age), the involuting phase (1-4 years of age), and the involuted phase (after four years of age) [2].

A detailed history and clinical examination are the keys to accurate diagnosis and differentiation between hemangiomas and vascular formations. The timing of appearance of the lesion after birth and rapid initial growth followed by gradual involution with residual scars point towards the diagnosis of hemangioma. However, there may be atypical clinical presentations of hemangiomas leading to confusion and a wrong diagnosis of vascular malformation (VM) due to similar clinical features and imaging findings. The author presents a rare case of Nicolau syndrome in an ear hemangioma following injection sclerotherapy leading to necrosis and full-thickness loss of skin and cartilage. This may be the first report of such a complication occurring in the ear, which was successfully reconstructed with a temporoparietal fascial (TPF) flap and conchal cartilage graft. A detailed literature review about the management of hemangiomas is discussed, emphasizing accurate clinical diagnosis and optimal treatment strategy.

## Case presentation

**Figure 1:**
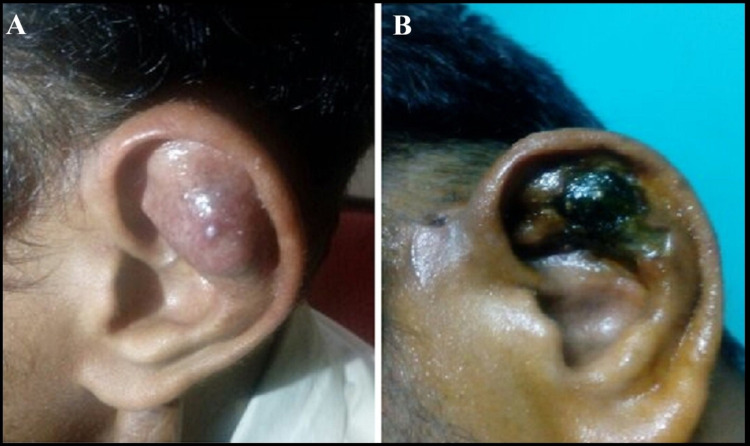
(A) Hemangioma of the superior and middle one-third of left ear involving the scaphal cartilage, antihelical fold, and conchal cartilage. (B) Early necrosis following biopsy and injection sclerotherapy of the lesion.

A 30-year-old male patient presented to the clinic with blackish discoloration of his left ear of one-month duration. The dermatologist in our hospital referred him. However, he had a history of vascular growth of the left ear, for which he underwent biopsy and injection sclerotherapy one month ago at another hospital. After a few days, he reported blackish discoloration with infection of the surrounding tissue of the affected ear (Figures 1A, 1B). 

**Figure 2:**
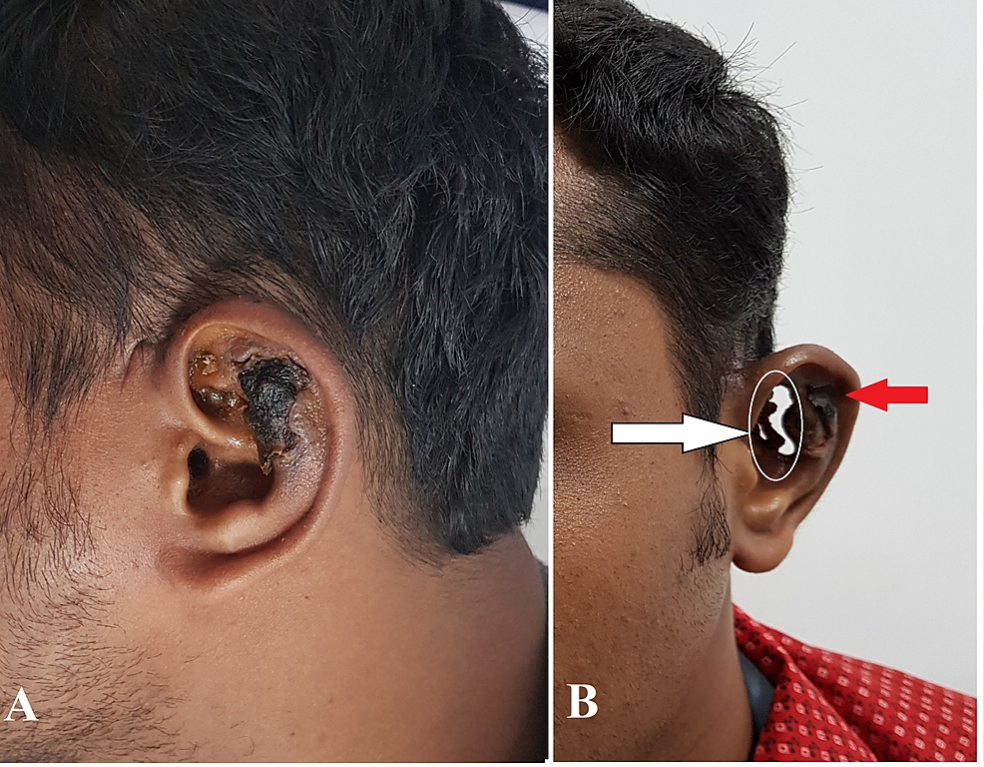
(A and B) Progression of necrosis of the affected ear ultimately leading to a full-thickness defect or “hole” in the conchal cartilage area (white arrow) and necrotic eschar over scaphal cartilage (red arrow).

On examination, there was necrosis over the superior and middle one-third of the ear involving the scaphal cartilage, antihelical fold, and conchal cartilage sparing the helical rim and the meatus (Figures 2A, 2B).

**Figure 3:**
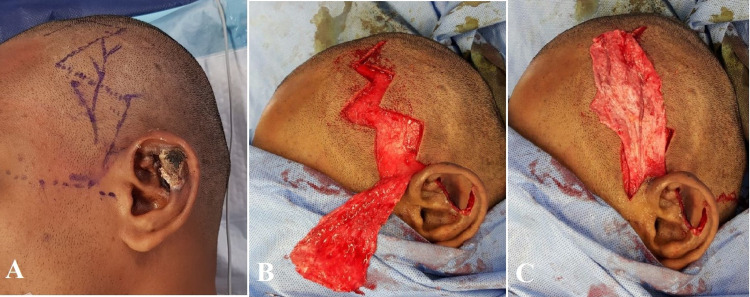
(A) Pre-operative marking of temporoparietal fascial (TPF) flap with dopplered vessels. (B and C) Harvest of TPF flap.

The previous biopsy record showed the lesion to be a hemangioma. As the necrosis was irreversible and well-demarcated, it was decided to proceed with excision of the necrotic tissue and reconstruct the defect with conchal cartilage from the contralateral ear for support, TPF flap for cover, and split skin graft for the outer lining. The outline of TPF flap was marked, the superficial temporal vessels dopplered and the flap harvested using a zig-zag incision (Figures 3A-3C).

**Figure 4:**
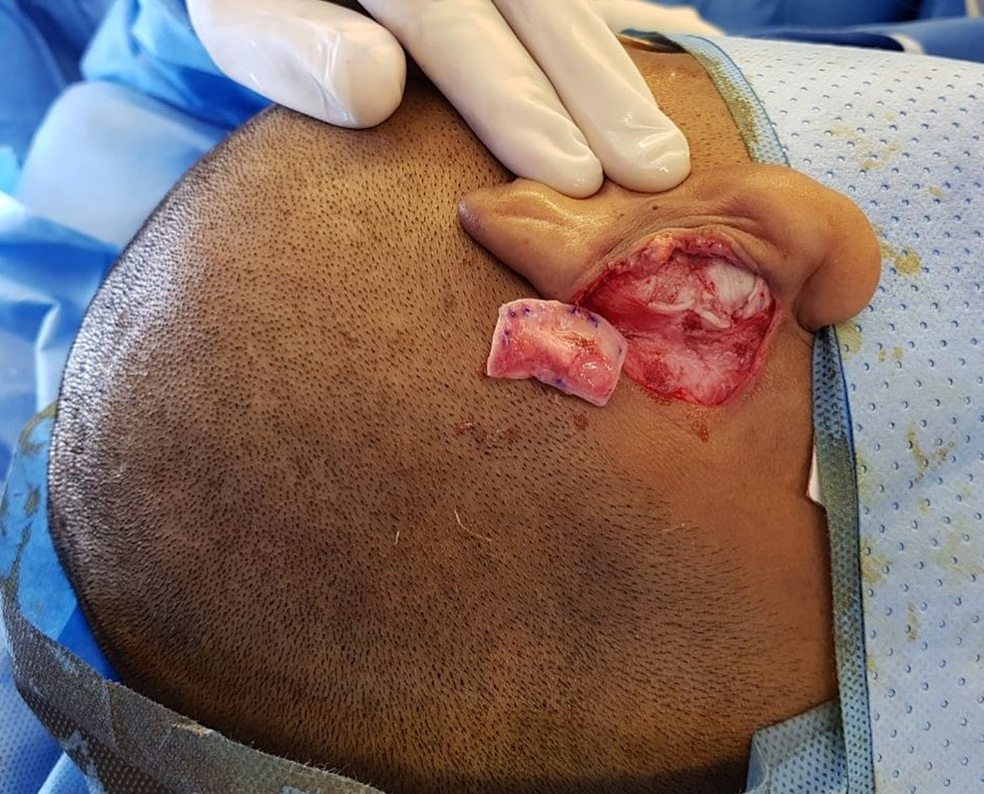
Harvest of the conchal cartilage graft from the opposite ear.

The conchal cartilage graft was harvested from the conchal bowl of the opposite ear (Figure 4).

**Figure 5:**
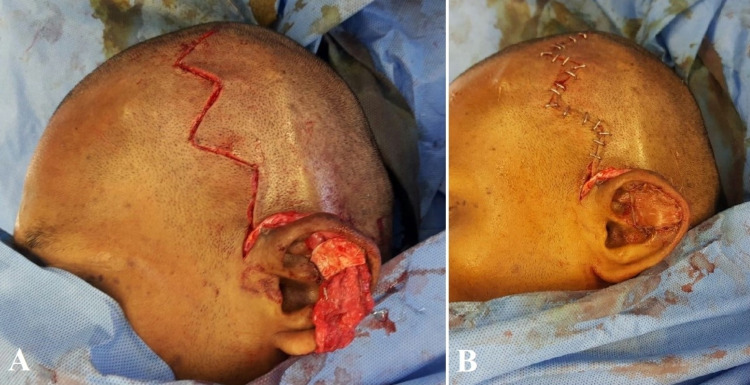
(A and B) TPF flap used to cover the cartilage graft and inset, followed by split skin graft for the final cover.

The TPF flap was turned over itself to cover the cartilage graft and the flap inset over the defect and final outer lining was provided by a split skin graft harvested from the thigh (Figures 5A, 5B).

**Figure 6:**
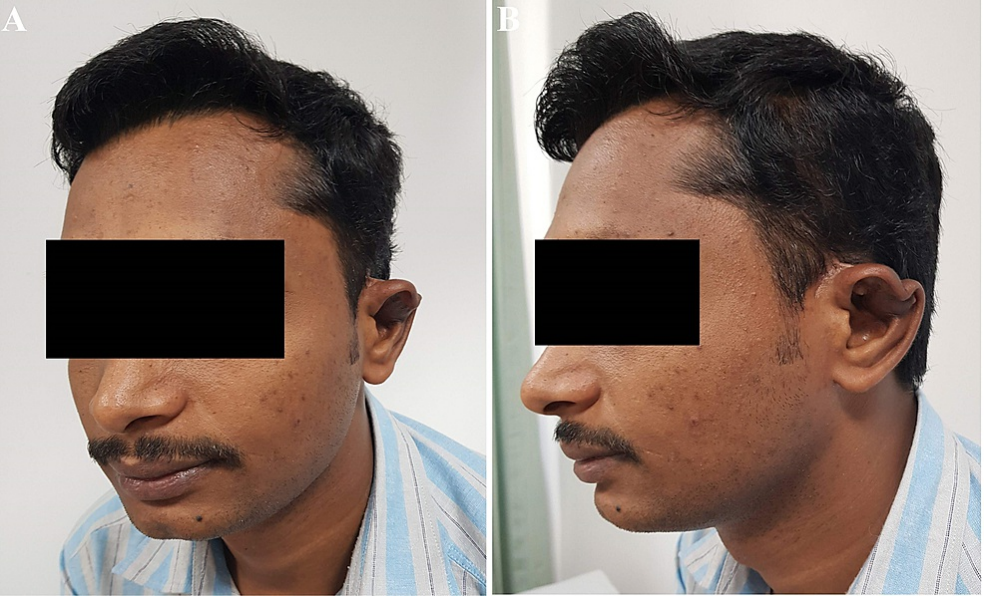
(A and B) Final outcome after six months.

The postoperative period was uneventful, and the biopsy confirmed the lesion to be a hemangioma. After surgery, he came for regular follow-up, and the outcome after six months is shown (Figures 6A, 6B).

He was satisfied with the postoperative result. However, there was postoperative notching, probably due to scar contracture at the helical margin. Therefore, a secondary scar revision (Z-plasty with/without full-thickness skin graft) was planned; unfortunately, he was lost to further follow-up after returning to his home country.

## Discussion

Most hemangiomas arise as a single, sporadic postnatal lesion with gradual onset of regression at 18 months. However, approximately half of these regressing hemangiomas leave behind residual lesions with permanent anatomical distortion, prominent scars, and altered skin pigmentation and texture. Unfortunately, atypical hemangiomas are mistaken for VMs because of the similarities in presentation and imaging properties. Two variants of atypical (congenital) hemangiomas rarely encountered include the rapidly involuting congenital hemangioma (RICH) and the non-involuting congenital hemangioma (NICH). These present as mature lesions at birth and may be mistaken for VMs. The NICH presents as a solitary, well-circumscribed, plaque-like lesion, does not undergo involution, instead grows with the child similar to a VM; diagnosis of this rare lesion will require histological studies and/or imaging studies [2].

Residual lesions were found in 69% of untreated cases in a 2011 study by Bauland et al. The main predictor for residual lesion was epidermal involvement, with no correlation being reported between growth pattern of hemangioma and risk of the residual lesion. Other studies reported a 50% incidence of residual lesions [3,4].

Hemangiomas are diagnosed clinically, but it is essential to do imaging studies to know the extent of soft tissue involvement and its flow characteristics when there is uncertainty. Therefore, MRI is the best imaging method for the diagnosis of hemangiomas. The tumor is isointense on T1, hyperintense on T2, and enhances during the proliferating phase. Ultrasound with duplex imaging is the initial modality of choice to assess hemangiomas as they are cheap, easily available, and non-invasive. They appear as a soft-tissue mass with the fast flow, decreased arterial resistance, and increased venous drainage. The only indication of angiography is if the patient is to undergo therapeutic embolization [2,5,6].

The management of hemangiomas with a conservative vis-à-vis surgical approach is a matter of debate. However, the natural history of hemangiomas, where most of these tumors undergo gradual regression followed by resolution, supports the traditional “wait and watch” policy. The guiding principles in the treatment of these tumors are to minimize complications that may lead to morbidity and mortality, improve physical appearance and psychosocial well being of the patient and family, and avoid overly aggressive procedures, which may lead to complications in hemangiomas with a high probability for excellent prognosis without intervention [7].

The options available for medical management of hemangiomas are varied and include corticosteroids, propranolol, interferon, lasers, and embolization.

Corticosteroids can be used either locally or systemically for the treatment of hemangiomas. The response to corticosteroid therapy is quite dramatic, as evidenced by growth arrest or rapid involution within the first week of initiation of therapy. Therefore, they are mainly indicated for use in small, isolated lesions in sensitive areas or large, complicated, life-threatening hemangiomas. However, in a study by Enjolras et al., 30% of study participants displayed accelerated involution following corticosteroid therapy, while 30% showed no response [4]. Nevertheless, corticosteroid therapy is still considered a first-line option in the medical management of hemangiomas due to their favorable therapeutic profile.

The serendipitous discovery of propranolol by Léauté-Labrèze et al. has brought about a paradigm shift in the management of proliferative hemangiomas. However, propranolol does not affect NICH and non-proliferative infantile hemangiomas [8]. It is now considered first-line therapy in the management of infantile hemangiomas and has superseded the role of corticosteroids. The recommended initial dosage of propranolol is 1 mg/kg/day divided thrice daily. The child should be monitored for bradycardia, which is the first sign of toxicity. The most common serious side effect of propranolol usage is hypoglycemia. It is most effective when started during the proliferative phase of hemangiomas (9-12 months of age) [2,8].

Recombinant interferon alpha-2a and alpha-2b is a second-line option in the medical management of hemangiomas. They are mainly indicated in large, disfiguring hemangiomas in sensitive areas and complicated, life-threatening tumors. However, interferon therapy must be initiated early and maintained for at least 15 months for maximum beneficial effect and to prevent rebound growth following abrupt cessation of therapy. It is essential to note that spastic diplegia may occur as a complication following interferon therapy [9,10].

Lasers cause photodisruption of tissues, which may help to arrest or regress the growth of hemangiomas. The pulsed dye laser (585 nm) can target vascular tissue and speed up ulcerated lesions' healing process. However, the penetration of pulsed dye laser is only 1 mm; hence it cannot be used in deep hemangiomas. The neodymium:yttrium-aluminum-garnet laser (Nd: YAG) can penetrate to 8 mm; however, its extensive effect on surrounding tissues can cause skin color, texture changes, and extensive scarring. The use of lasers in managing hemangiomas is debatable. Still, there are specific scenarios where it may be beneficial, such as an early therapeutic option during infancy, large hemangiomas located in unfavorable anatomical regions, and the treatment of residual lesions after involution [11,12].

There have been only isolated case series and reports about the use of sclerosants to treat hemangiomas. Therefore, it is not considered a first-line option because of the high risk of complications. However, it has been traditionally used in VMs, especially - low flow VMs, venous and lymphatic malformations. In the case of hemangiomas, sclerosants are mainly used as part of embolization therapy for large, life-threatening hemangiomas, usually multifocal hepatic lesions, that can cause high-output congestive heart failure. However, sclerotherapy for hemangiomas can lead to more scarring in the long term [13,14].

A rare and dreaded complication associated with injection sclerotherapy is Nicolau syndrome, leading to variable degrees of necrosis of skin and underlying soft tissue. This manifests initially as skin color changes, severe pain, numbness, and paresthesia. If missed, frank skin ulceration and necrosis can progress, leading to irreversible tissue damage and permanent scarring [15,16]. Nevertheless, injection sclerotherapy is a well-established option for treating varicose veins, spider veins, hemorrhoids, hydroceles, and vascular malformations. In addition, there are reports of the use of sclerotherapy in pyogenic granuloma with promising results [17,18].

In our case report, the patient had ear necrosis following injection sclerotherapy for a residual ear hemangioma leading to Nicolau syndrome and consequently full-thickness loss of skin and cartilage of the upper and middle thirds of the ear. This is the first report of Nicolau syndrome following sclerotherapy for an ear hemangioma to the best of our knowledge. This complication has also been reported after sclerotherapy in a pyogenic granuloma [19].

The complications seen following injection sclerotherapy are mainly due to accidental extravasation into the surrounding tissue. The collateral damage following extravasation of sclerosant can be minimized by ligating the stalk of pedunculated lesions, pressure along the lesion's periphery, tourniquet use in extremities, cyanoacrylate, or balloon embolization before sclerotherapy. In addition, certain medications, if initiated early, can reverse the side effects of sclerosant extravasation, and these include anticlotting drugs (heparin), vasodilator drugs (pentoxifylline, topical nitroglycerine), systemic corticosteroids, and non-steroidal anti-inflammatory drugs (NSAIDs) to reduce inflammation and local wound care. Injection sclerotherapy is better avoided in vascular lesions on fingers and toes, ear, nose tip, penile tip (end arteries), and children below 18 years [17,19].

Finally, the traditional approach of “masterly inactivity” in the management of hemangiomas is still relevant today because of the tendency of hemangiomas to involute with time. Surgery during the proliferative phase is not recommended due to the possibility of excessive bleeding and poor visualization. Instead, surgery is mainly indicated for the removal of residual fibrofatty tissue and scar. However, when large, complicated, and life-threatening hemangiomas in sensitive areas cause obstructive symptoms (loss of vision, airway compromise, and feeding difficulty), early surgical intervention is warranted. Surgical intervention is also sometimes indicated for large, prominent lesions without functional compromise, but for aesthetic reasons to minimize the psychological issues faced by the growing child and parents. Late surgery is done only after completing the involution phase and is mainly indicated to correct abnormal contour and anatomical distortion caused by residual scarring and fibrofatty tissue [20].

## Conclusions

This article highlights the importance of proper clinical diagnosis of hemangiomas and discusses common treatment strategies to optimize patient outcomes with particular emphasis on minimizing complications. There should be an initial wait and watch approach in managing hemangiomas with a low threshold for medical therapy and a high threshold for surgery. Surgery should be reserved for involuted hemangiomas and those associated with severe life-threatening complications.

The role of injection sclerotherapy in managing hemangiomas is debatable, and further studies are warranted before definitive conclusions can be made regarding the precise indications for this modality. Furthermore, judicious patient selection with informed written consent and counseling are advised to avoid complications and medico-legal issues.
